# A case report and literature review of proton pump inhibitors inducing insulin autoimmune syndrome

**DOI:** 10.3389/fendo.2026.1784299

**Published:** 2026-06-03

**Authors:** Wenrui Zhang, Jiayan Wen, Siming Qi, Guohui Zhang, Xubin Yang, Longyi Zeng, Wen Xu, Hongrong Deng

**Affiliations:** 1 Department of Cardiovascular and Metabolic Medicine, Zhuhai Xiangzhou District People’s Hospital, Zhuhai, Guangdong, China; 2 Department of Endocrinology and Clinical Nutrition, The Fifth People’s Hospital of Shunde District, Foshan, Guangdong, China; 3 Department of Endocrinology and Metabolism, Heshan People’s Hospital, Heshan, Guangdong, China; 4 Geriatrics Department, Shenzhen Bao’an District People’s Hospital, Shenzhen, Guangdong, China; 5 Department of Endocrinology and Metabolism, Third Affiliated Hospital of Sun Yat-sen University, Guangdong Provincial Key Laboratory of Diabetology, Guangzhou, Guangdong, China

**Keywords:** Clopidogrel, hypoglycemia, insulin antibody, insulin autoimmune syndrome, proton pump inhibitor

## Abstract

**Background:**

Insulin autoimmune syndrome (IAS) is an autoimmune endocrine disorder characterized by hyperinsulinemia, positive insulin autoantibodies (IAA), and hypoglycemia. IAS was previously thought to be induced by drugs containing thiol groups.

**Objective:**

We describe a case highlighting that IAS may be temporally associated with a proton pump inhibitor (PPI) exposure. This article reports a patient who took beraprost sodium, clopidogrel, atorvastatin calcium, and the PPI pantoprazole and was subsequently diagnosed with IAS, presenting with a high IAA level, refractory hypoglycemia, and markedly elevated insulin levels. The patient’s hypoglycemic episodes were ultimately relieved after discontinuation of the PPI, accompanied by a gradual decrease in the IAA level to the normal range.

**Key findings:**

HLA analysis revealed HLA-DRB1*0403, DQB1*0302, and DQA1*0301, indicating the patient’s genetic susceptibility to IAS, which was possibly triggered by the PPI.

**Clinical relevance:**

This case report may alert clinicians to possible PPI-associated IAS, helping to avoid misdiagnosis and enable patients to receive timely treatment.

## 1 Introduction

Hypoglycemia accompanied by hyperinsulinemia suggests either endogenous or exogenous insulin excess. After excluding hypoglycemia due to glucose−lowering medications, the diagnosis of insulinoma is first established on clinical and biochemical grounds, most notably through a supervised fasting test demonstrating inappropriately elevated insulin levels during hypoglycemia, followed by imaging studies for tumor localization (1).

In patients presenting with hyperinsulinemic hypoglycemia, markedly elevated insulin levels with non−suppressed C−peptide, and positive insulin autoantibodies (IAA), insulin autoimmune syndrome (IAS) should be considered early to avoid misdiagnosis as insulinoma (2). IAS is characterized by recurrent episodes of hypoglycemia resulting from high concentrations of immunoreactive insulin bound to circulating autoantibodies. The pathogenesis involves genetic predisposition, immune dysregulation, and drug exposure. The most common triggers are medications containing thiol groups, such as methimazole, which can induce structural changes in insulin and promote autoantibody formation. Drugs that generate thiol metabolites, including clopidogrel and α−lipoic acid, have also been implicated. Rare case reports have suggested a possible association between IAS and proton pump inhibitors (PPIs), although these agents neither contain nor generate thiol groups, and the strength of this association remains weak (3).

This article reports a rare case of severe hypoglycemia in an elderly patient who was diagnosed with IAS, possibly associated with the PPI pantoprazole.

## 2 Case description

### 2.1 Presenting symptoms

The patient was admitted to Heshan People’s Hospital on June 18, 2024, due to “repeated sweating, palpitations, blurred vision, and fatigue for one month”. When these symptoms occurred, he experienced marked hunger, and the symptoms were relieved by eating. The patient measured his fingertip blood glucose level at 2.7 mmol/L before food intake. He also reported a weight gain of 2.5 kg over 1 month. Although oral glucose tolerance test (OGTT) is not a standard test for hypoglycemia evaluation, it showed detailed blood glucose levels (0, 0.5, 1, 2, and 3 h blood glucose levels were 4.32, 11.62, 15.64, 15.85, and 3.91 mmol/L, respectively). However, the insulin and C-peptide release test (dynamic test performed at Heshan People’s Hospital, electrochemiluminescence assay) showed disproportionately elevated insulin levels (fasting insulin was 804 mU/L, and stimulated insulin levels were >1,000 mU/L) relative to C−peptide (0, 0.5, 1, 2, and 3 h C-peptide were 2.12, 5.86, 8.65, 10.45, and 6.05 nmol/L), consistent with assay interference due to insulin autoantibodies. In addition, no lesion was identified on chest and whole-abdominal CT imaging, making the diagnosis unclear. Therefore, he was transferred to our hospital.

### 2.2 Demographic and psychosocial data

The patient was a 67-year-old male retired worker, who had always lived in Heshan City, Guangdong Province, China, with no psychosocial problems.

### 2.3 Past medical history and medications

On April 9, 2024, he underwent arterial balloon dilatation and stenting of the lower extremities by iodine contrast imaging. After the operation, he began taking “beraprost sodium (40 μg tid), clopidogrel (75 mg qd), atorvastatin calcium (20 mg qn), and pantoprazole (40 mg qd)”. He denied a medical history of diabetes, coronary heart disease, thyroid disease, malignant tumor, etc. No direct exposure to classical thiol−containing drugs, although clopidogrel (a prodrug generating thiol metabolites) was used.

### 2.4 Physical examination

The patient exhibited no obvious abnormalities in general appearance. His vital signs were stable, with normal blood pressure. Anthropometric measurements revealed a height of 165 cm, a weight of 52 kg, and a BMI of 19.1 kg/m². Physical examination showed no lower-extremity edema, and dorsalis pedis artery pulsations were intact. All other systemic examinations were unremarkable.

### 2.5 Laboratory and imaging findings

Routine laboratory tests, including blood count, urinalysis, stool examination, liver and kidney function, electrolytes, erythrocyte sedimentation rate, tumor markers, hepatitis screening, and catecholamine metabolites, showed no significant abnormalities. Hormonal parameters (cortisol, FT3, FT4, TSH, and IGF−1) and diabetes−related autoantibodies (IAA, ICA, GADA, and TPA) were assessed using chemiluminescence immunoassays. The patient exhibited elevated insulin and C−peptide levels with normal fasting glucose and disproportionately elevated insulin level relative to C−peptide. HbA1c was 6.2%. The IAA level was markedly elevated, while other autoantibodies were within the normal range. The main laboratory findings from our hospital are summarized in **Table 1**, including retested insulin and C-peptide levels (chemiluminescence assay). The interference testing (PEG precipitation, dilution linearity, and heterophile blocking) was not performed, which may affect insulin testing due to the presence of IAA.

**Table 1:** Laboratory findings in our hospital.

Item	Result	Reference ranges
AST(U/L)	35	15-40
ALT(U/L)	44	3-35
Creatinine (μmol/L)	78.0	31.8-116.0
Cortisol (8AM, nmol/L)	375.51	117.75-686.85
IGF-1 (ng/mL)	110	116-368
FT3 (pmol/L)	5.21	3.85-6.3
FT4 (pmol/L)	15.33	12.8-21.3
TSH (mU/L)	1.36	0.75-5.6
Fasting glucose (mmol/L)	4.19	3.90-6.10
Fasting C-peptide (nmol/L)	1.45	0.27-1.28
Fasting insulin (mU/L)	75.20	3-25
HbA1c (%)	6.2	<6.0
IAA(COI)	115.57	<0.9
ICA(COI)	0.14	<0.9
GADA(U/mL)	0.81	<10
TPA(U/mL)	0.92	<10

AST, aspartate transaminase; ALT, alanine transaminase; IGF-1, insulin-like growth factor-1; FT3, free triiodothyronine; FT4, free thyroxine; TSH, thyroid stimulating hormone; HbA1c, glycated hemoglobin A1c; IAA, insulin autoantibody; ICA, islet-cell antibody; GADA, glutamic acid decarboxylase antibody; TPA, tyrosine phosphatase antibody.

**Figure 1:**
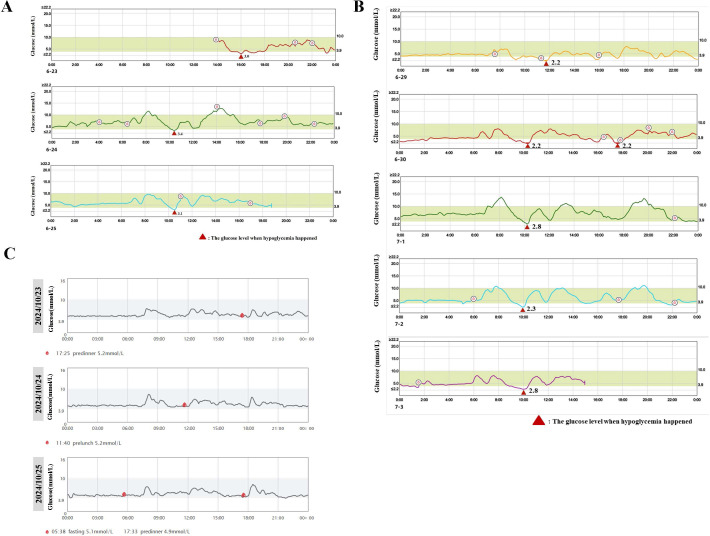
The patient’s glucose levels in the continuous glucose monitoring system (CGMS). **(A)**, glucose levels before stopping clopidogrel in CGMS in our hospital; **(B)**, glucose levels while taking prednisone after stopping clopidogrel in CGMS in our hospital; **(C)**, glucose levels after stopping pantoprazole and prednisone in CGMS during follow-up. We can see hypoglycemia episodes in **(A, B)**, not in **(C)**.

**Figure 2:**
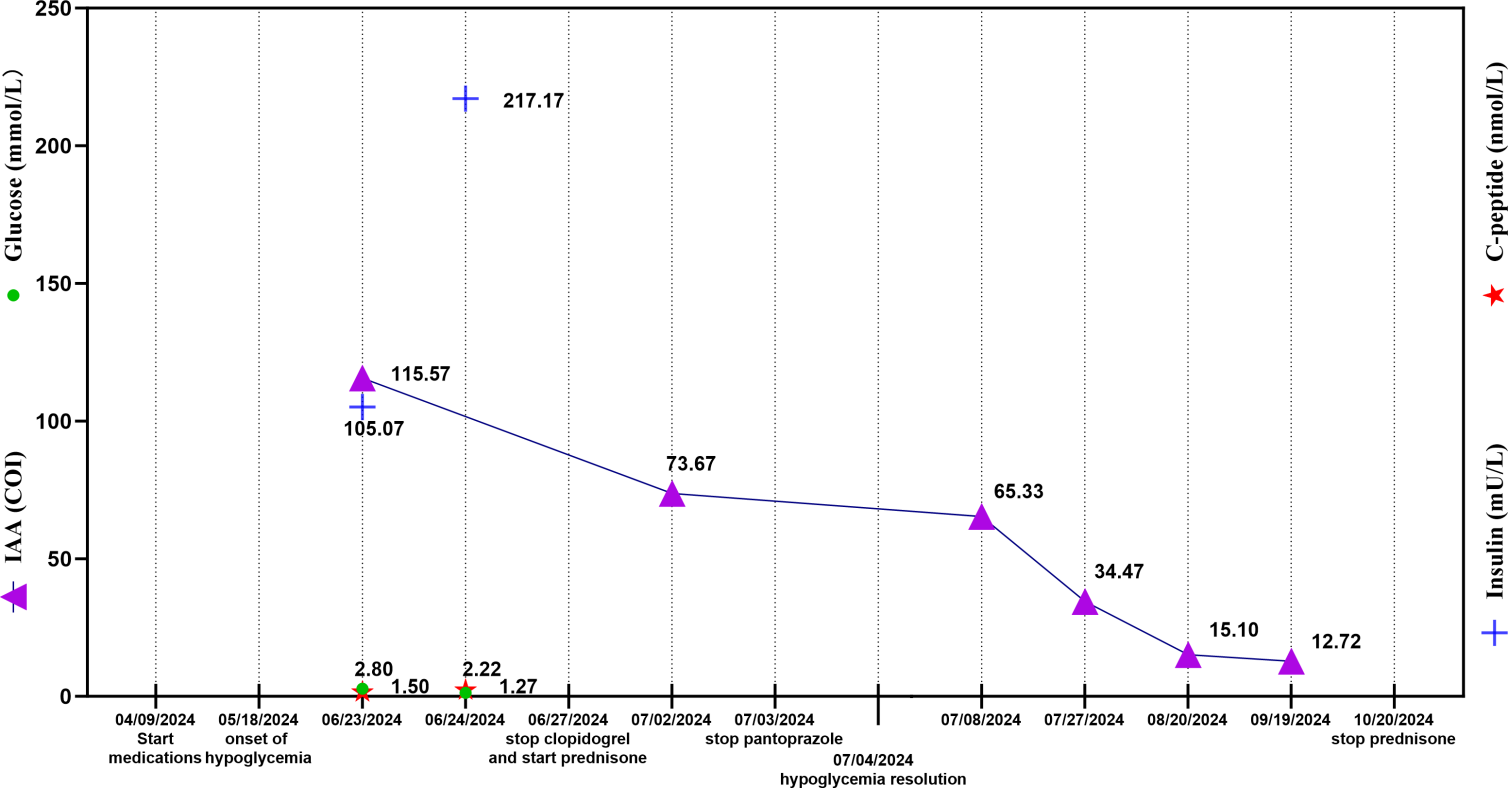
The timeline of the diagnostic and treatment process of our patient. The sequence of medication changes, hypoglycemic episodes, laboratory findings, and insulin autoantibodies (IAA) titers are summarized in this timeline image, showing start and stop dates of pantoprazole, clopidogrel, and other relevant drugs, the onset and resolution of hypoglycemia, key laboratory values (glucose, insulin, C-peptide during hypoglycemic episodes on June 23 and June 24, and IAA change process), and major interventions with follow-up milestones. “Start medications” means the patient started “beraprost sodium (40 μg tid), clopidogrel (75 mg qd), atorvastatin calcium (20 mg qn), and pantoprazole (40 mg qd)”.

Continuous glucose monitoring system (CGMS) data indicated that the patient still experienced repeated hypoglycemia despite frequent eating and a low-carbohydrate, high-fat, high-quality protein diet (**Figure 1A**). During hypoglycemic episodes in the hospital, his plasma glucose levels were 2.80 and 1.27 mmol/L, accompanied by markedly elevated C-peptide and insulin levels (**Figure 2** shows the glucose, insulin, and C-peptide levels during hypoglycemic episodes on June 23 and June 24). Upper abdominal MRI showed small cysts in liver segments S2 and S4, with mild dilation of the intrahepatic bile ducts and no lesions in the pancreas. No definite hypermetabolic tumor signs were detected on fluorine-18-fluorodeoxyglucose (18F-FDG) positron emission tomography/computed tomography (PET/CT) or gallium-68 (68Ga)-somatostatin analog (SSA) PET/CT imaging.

## 3 Differential diagnosis and reasoning

### 3.1 IAS

The patient had a clear diagnosis of hypoglycemia, with elevated insulin and C-peptide levels, no use of oral hypoglycemic drugs or insulin, and no evidence of other endocrine hormones affecting blood glucose. Given his elevated IAA, IAS was highly suspected.

### 3.2 Insulinoma or multiple endocrine neoplasia

Levels of IGF-1, growth hormone, cortisol, etc., were normal. Abdominal CT did not indicate pancreatic or other tumor lesions, nor did MRI. No definite hypermetabolic tumor signs were detected on ^18^F-FDG and 68Ga-SSA PET/CT imaging. In addition, our patient’s insulin levels were extremely high despite the short duration of hypoglycemia. Insulinoma was considered unlikely given negative imaging and the presence of insulin autoantibodies. There was no clinical or biochemical evidence suggestive of multiple endocrine neoplasia (MEN) syndrome.

### 3.3 Reactive postprandial hypoglycemia

A 2-h glucose level >11.1 mmol/L on OGTT and an HbA1c of 6.2%, together with hypoglycemia, might suggest reactive postprandial hypoglycemia in prediabetes; however, the rather high insulin level and rather low glucose level during hypoglycemic episodes argued against this diagnosis.

### 3.4 Other causes of hypoglycemia

Other causes of hypoglycemia, including factitious hypoglycemia and non-islet cell tumor hypoglycemia, were considered unlikely based on clinical and laboratory findings.

### 3.5 Final diagnosis

IAS, possibly induced by a recently used drug.

## 4 Treatment and follow-up

Because IAS can be induced by clopidogrel, we discontinued clopidogrel on June 27, 2024, and replaced it with oral aspirin. Dietary modification with frequent small meals was maintained. We also began treatment with prednisone (10 mg tid). After 3 days, the patient still had frequent hypoglycemia. Therefore, we added acarbose (0.1 g tid) and changed prednisone to 40 mg qd. Although the IAA level decreased to 73.67 COI, our treatment could not prevent hypoglycemia before lunch until July 3, 2024 (**Figure 1B** shows that he still experienced hypoglycemia every day on CGMS). After a literature review, we found a few case reports suggesting that PPIs were possible triggers for IAS. On July 3, 2024, we discontinued pantoprazole. Rapid hypoglycemia resolution following discontinuation raises the possibility that PPI may be associated with IAS. Clopidogrel was considered less likely, although its role cannot be completely excluded. Although we attempted to restart clopidogrel, the patient refused. His IAA level decreased to 65.33 COI after 5 days. We discontinued acarbose and continued prednisone alone (10 mg tid) for IAS, after which he was discharged. Subsequently, the patient’s human leukocyte antigen (HLA) typing showed DRB1 * 0403/0901, DQB1 * 0302/0303, DQA1 * 0301/0302, and DRB4 * 0103. We then gradually tapered the prednisone dose.

## 5 Outcomes

On September 19, 2024, his IAA level decreased to 12.72 COI (**Figure 2** shows details of the IAA changes). On October 15, 2024, the patient’s IAA level returned to normal (12.087 AU/mL, tested at another hospital; reference range, 0–20; chemiluminescence assay; Shenzhen New Industries Biomedical Engineering Co., Ltd). Although the patient refused to undergo repeat OGTT and HbA1c testing, his own CGMS indicated normal blood glucose levels after prednisone was discontinued on October 20, 2024, following 3 months of treatment (**Figure 1C** shows his CGMS glucose levels). The patient has not experienced hypoglycemia again to date. The patient was generally satisfied with the treatment outcome but is currently unwilling to undergo a rechallenge with clopidogrel. **Figure 2** shows the timeline of the diagnostic and treatment processes.

## 6 Literature review

Our search was performed using the following online databases: PubMed, Web of Science, Scopus, China National Knowledge Infrastructure, and Chinese Medical Journal Full-text Database, with the keywords “insulin autoimmune syndrome” or “Hirata disease” or “Hirata’s disease” or “Hirata syndrome” or “insulin autoimmune” and “pantoprazole” or “omeprazole” or “esomeprazole” or “rabeprazole” or “lansoprazole” or “ilaprazole” or “proton pump inhibitor”. The search period ranged from January 1972 to the present. One case involving proton pump inhibitors and oxaliplatin chemotherapy was excluded (4), because the hypoglycemia may have been induced by a synergistic interaction between these two drug classes. Based on our search strategy, six detailed case reports describing a possible association between PPIs and IAS were identified (5–10). Although the number of cases is limited, some recurring features can be observed. Reported cases originate from diverse geographic regions, including Asia, the United States, and Europe, consistent with known ethnic susceptibility patterns (3, 11, 12). No clear sex predominance can be established due to the small sample size. The interval between PPI exposure and onset of hypoglycemia ranged from 2 weeks to 2 months. IAA levels were positive in all tested patients, as expected for the diagnosis of IAS. The characteristics of these six cases are summarized in **Table 2**.

**Table 2:** A literature review of detailed case reports on proton pump inhibitor-associated IAS.

Patients	Age, year	Sex	Country	Suspected cause	Time to cause hypoglycemia	Insulin autoantibody	Insulin level (mU/L)	High insulin, unsuppressed C-peptide	IAS-related HLA loci	Associated autoimmune diseases	Therapy
1 (5)	72	Male	India	Pantoprazole	Not mentioned	Not mentioned	123	Yes	DRB1*0403, DRB1*0404	Rheumatoid factor and antinuclear antibodies were positive	Pantoprazole was discontinued
2 (6)	65	Female	The United States	Omeprazole	Not mentioned	Positive	202	Yes	DRB1*03, DRB1*14, DRB3*02	Not reported	Omeprazole was switched to an H2 receptor blocker
3 (7)	56	Female	China	Esomeprazole	2 weeks	Positive	237.7	Yes	Not determined	None	Esomeprazole was discontinued and administering acarbose
4 (8)	58	Male	China	Esomeprazole	4 weeks	Positive	5940	Yes	Not determined	None	Esomeprazole was discontinued
5 (9)	67	Male	Sri Lanka	Omeprazole	4 weeks	Positive	>6000	Yes	Not determined	None	Omeprazole was discontinued and administering prednisone
6 (10)	27	Male	China	Omeprazole	Not clear (short time)	Positive	173	Yes	DRB1*0406	None	Omeprazole was discontinued

Most cases did not report concomitant autoimmune diseases, although IAS is classically associated with autoimmune conditions. HLA analyses, when available, identified alleles such as HLA-DRB1*0403 and DRB1*0406 (5, 10), consistent with previously reported genetic susceptibility patterns (11, 12). Although the American patient’s results, HLA-DRB1*03, DRB3*02, and DRB1*14, have not been reported in Hirata syndrome, the author suggested that the genetic spectrum of IAS may be more heterogeneous (6). The suspected triggers for IAS were different PPIs, including omeprazole in three patients, esomeprazole in two patients, and pantoprazole in one patient. In all reported cases, hypoglycemia improved after discontinuation of the PPI, although causality cannot be definitively established. Given the very small number of cases, these observations should be interpreted with caution.

## 7 Discussion

IAS, also known as autoimmune hypoglycemia, was first reported by the Japanese scholar Hirata in 1972 (13). IAS results from the formation of IAA characterized by low affinity and high binding capacity, leading to postprandial insulin sequestration and delayed dissociation, which causes hypoglycemia (3, 12, 14). Scatchard analysis demonstrates the low-affinity, high-capacity binding characteristics of IAA (15, 16). When insulin is released from IAA, severe hypoglycemia can occur, insulin levels are disproportionately elevated relative to C−peptide (10, 17). In our case, the endogenous hyperinsulinemic hypoglycemia, markedly elevated insulin levels (>100 mU/L) with non−suppressed C−peptide, and positive IAA were all consistent with IAS. In addition to his high IAA level, our patient had no history of exposure to hypoglycemic agents (including insulin). While the overall findings supported a diagnosis of IAS, insulinoma could not be entirely ruled out (18).

**Figure 3:**
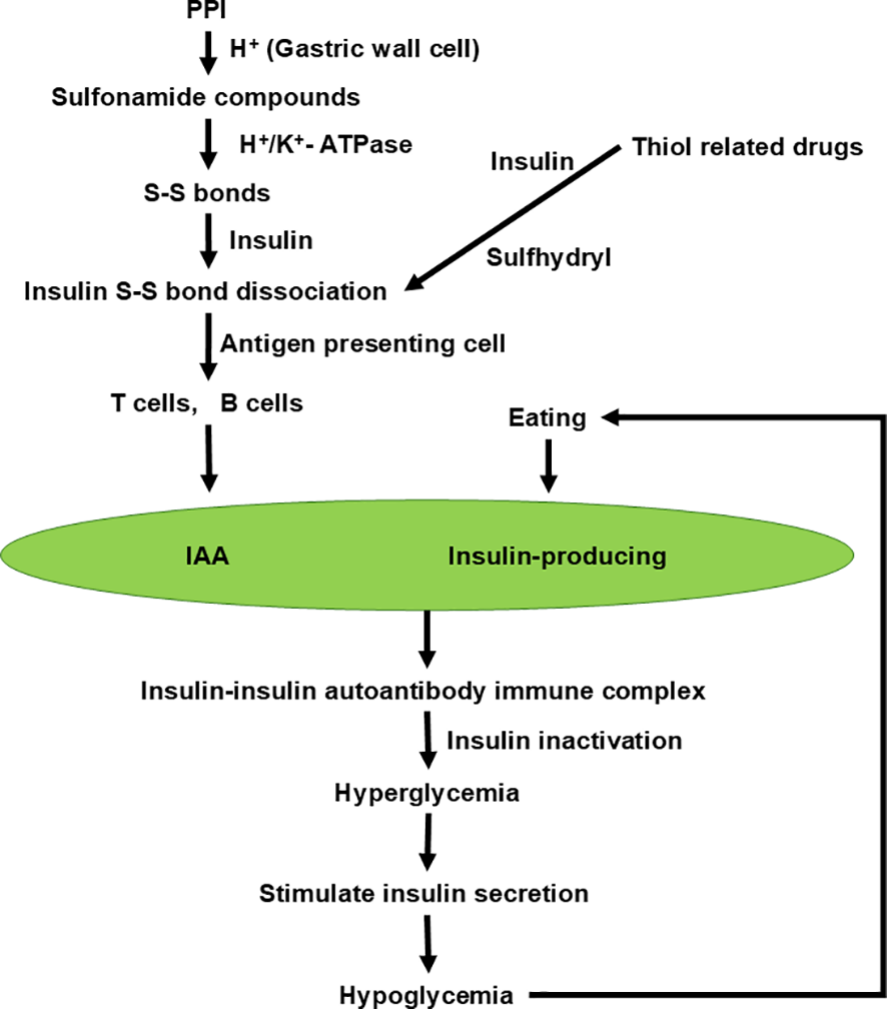
The proposed mechanism underlying IAS potentially associated with PPIs or thiol-related drugs. IAS, insulin autoimmune syndrome; PPIs, proton pump inhibitors; IAA, insulin autoantibodies.

Patients with IAS often have other autoimmune diseases, and the use of thiol-containing drugs is one of the main triggering factors. It has been hypothesized that thiol compounds may interact with disulfide bonds in insulin, altering the chemical and immune properties of insulin and making it more immunogenic, thereby leading to changes in endogenous insulin structure, activation of self-antigens, production of IAA, and ultimately hypoglycemia (12, 19). More than 50% of cases are related to the use of methimazole (20), due to its sulfhydryl groups. In recent years, other drugs without thiol groups have also been found to induce IAS, such as clopidogrel. Although clopidogrel itself does not contain sulfhydryl groups, it becomes a biologically active compound after metabolism by cytochrome P450 enzymes, and its active metabolites contain sulfhydryl groups that can cause IAS (18, 21, 22). The mechanism is similar to that of sulfhydryl-containing drugs. Our patient was treated with both clopidogrel and a PPI, whereas PPIs have only rarely been reported to cause IAS (10). In this case, clopidogrel was a potential confounder when we explored the triggering factors of IAS. However, discontinuation of clopidogrel together with corticosteroid treatment was ineffective after 6 days, and the patient still experienced frequent hypoglycemic episodes. Therefore, we suspected that clopidogrel may not be the cause of his IAS. Thereafter, the hypoglycemia completely resolved within 24 h after discontinuation of the PPI (pantoprazole). The temporal association supports a possible relationship but does not establish causality. PPIs are weakly basic benzimidazole derivatives that can rapidly penetrate the gastric parietal cell membrane, accumulate in strongly acidic secretory canaliculi, convert into sulfonamide compounds, and covalently bind to the thiol group of H+/K+-ATPase to form disulfide bonds (23, 24). The mechanism underlying the association between PPIs and IAS remains unclear. It has been hypothesized that reactive metabolites could interact with insulin or immune pathways, but this has not been experimentally confirmed (9, 10) (**Figure 3** illustrates the proposed mechanism by which PPIs and thiol-related drugs may trigger IAS). Clopidogrel, captopril, omeprazole, esomeprazole, methimazole, and pantoprazole have been reported in pharmacovigilance analyses as potential signals for IAS (25), which may support the potential relevance of our findings.

**Table 2** shows that five of the six detailed reported cases (5–10) met all the clinical diagnostic criteria, including hyperinsulinemic hypoglycemia, markedly elevated insulin levels (>100 mU/L) with non−suppressed C−peptide, and positive IAA (18). The patient from India also took pantoprazole and clopidogrel simultaneously; the physician chose to discontinue pantoprazole, and his hypoglycemic symptoms gradually subsided (5). One patient from China developed IAS after taking esomeprazole for 2 weeks but did not develop hypoglycemia after taking omeprazole for 1 month (7). From these few cases, we found that the time to onset of hypoglycemia may differ among different PPIs; therefore, the lack of IAS during omeprazole use in that patient may have been due to the short duration of exposure. Like our patient, an extended OGTT with 75 g of glucose showed paradoxical hyperglycemia followed by a rapid decline in blood glucose, accompanied by very high insulin levels (5, 7, 10). Markedly elevated or disproportionate insulin levels may raise suspicion for IAS. In this patient, blood glucose levels briefly increased and then rapidly decreased within 3 h during OGTT. Combined with a slightly elevated HbA1c, these findings could easily be mistaken for reactive postprandial hypoglycemia. There has even been a case report in which IAS was misdiagnosed as diabetes and treated with a sulfonylurea (10). Remission during follow-up in our patient reminds clinicians to recognize the hyperglycemic state induced by loss of insulin efficacy and frequent food intake. All six patients gradually became free of hypoglycemic episodes after discontinuation of the PPI, while one patient received acarbose and another received prednisone. The treatment of IAS includes discontinuation of suspected drugs, improvement of dietary structure, glucose supplementation, and the addition of glucocorticoids when necessary (18). Recently, drugs such as rituximab and somatostatin have also been reported to be effective, and in severe cases, plasma exchange can be performed (26, 27). Many patients experience spontaneous remission (18), and IAA may become negative within 6 months (17). After pantoprazole was discontinued, follow-up of our patient showed that the IAA level gradually returned to normal within 4 months, and prednisone was discontinued without relapse of hypoglycemia. This case reminds us that clear identification of the suspected drug may be useful to avoid overtreatment.

At present, the majority of reported IAS cases are from Japan and China, significantly more than those from Europe and America (12), suggesting racial susceptibility. HLA-DR4 has been considered an important susceptibility locus for IAS (13). A literature review published in 2024 summarized HLA analyses from 68 patients with IAS (28), DRB1*0406 (26 cases, primarily found in East Asian countries) and DRB1*0403 (18 cases, predominantly in non-East Asian countries) were the most frequently reported. Moreover, the key locus in the Caucasian population is DRB1*0403 (11). HLA-DRB1*0403 has been reported to be associated with clopidogrel-induced IAS in China (22). Although HLA-DRB1*0403 is reported in 75% of cases associated with clopidogrel, its broad geographic distribution (documented in China, France, and USA) contrasts sharply with the region-specific drug–allele associations observed with methimazole and α-lipoic acid (22, 28). However, DRB1*0403 was also found in the pantoprazole-associated case from India (5). Therefore, DRB1*0403 may be associated not only with clopidogrel-induced IAS but also with other drugs associated IAS, such as pantoprazole in our patient. In addition to DRB1*0406 and DRB1*0403, IAS has also been associated with DQA1 * 0301, DQB1 * 0302, DRB1 * 0415, and DRB1 * 1301 (17). The HLA analysis results of our patient included HLA-DRB1 * 0403, DQB1 * 0302, and DQA1*0301, indicating that the occurrence of IAS in this patient was not random and that he had genetic susceptibility.

We must acknowledge the limitations of pharmacovigilance data in collecting adverse drug reactions, including reporting bias, incomplete comedication information, and weak signal-level evidence. The patient was also taking clopidogrel, a known trigger of IAS and other autoimmune reactions. Although the Naranjo score, a standardized 10−item algorithm used to estimate the likelihood that an adverse event is drug−related, indicated a possible association between both drugs (clopidogrel and pantoprazole) and the IAS, the resolution of hypoglycemia following the discontinuation of the PPI points to the drug as a potential contributing factor. The other two drugs “beraprost sodium and atorvastatin calcium” he is taking, as well as the iodine used in lower limb arterial surgery angiography, do not contain thiol groups and do not produce thiol groups during metabolism. Moreover, there have been no reports of their association with hypoglycemia, so the likelihood of their association with IAS is extremely low. No recurrence during follow−up represented a favorable outcome. The mechanism of PPI may trigger IAS remains hypothetical, and causality cannot be proven in several cases. Basic research is still needed to verify the mechanism of PPI-associated hypoglycemia.

PPIs are widely used in clinical practice worldwide, including in Europe and America, for both therapeutic and preventive purposes (29, 30). This article reports in detail a case of IAS possibly caused by a PPI, which should draw the attention of clinicians and researchers. Based on the case we report and the six previously published cases, PPIs are implicated as possible triggers for IAS, although the mechanism remains unclear. Accurate diagnosis of IAS and identification of the causative drug may be useful to avoid misdiagnosis in clinical practice and to enable patients to receive timely treatment.

## Data Availability

The original contributions presented in the study are included in the article/supplementary material. Further inquiries can be directed to the corresponding authors.
